# Pleiotropic cellular responses underlying antibiotic tolerance in *Campylobacter jejuni*

**DOI:** 10.3389/fmicb.2024.1493849

**Published:** 2024-11-22

**Authors:** Eunshin Cho, Jinshil Kim, Jeong In Hur, Sangryeol Ryu, Byeonghwa Jeon

**Affiliations:** ^1^Department of Food and Animal Biotechnology, Research Institute of Agriculture and Life Sciences, Seoul National University, Seoul, Republic of Korea; ^2^Department of Agricultural Biotechnology, Seoul National University, Seoul, Republic of Korea; ^3^Center for Food and Bioconvergence, Seoul National University, Seoul, Republic of Korea; ^4^Department of Food Science and Biotechnology, Carbohydrate Bioproduct Research Center, Sejong University, Seoul, Republic of Korea; ^5^Division of Environmental Health Sciences, School of Public Health, University of Minnesota, St. Paul, MN, United States

**Keywords:** *Campylobacter jejuni*, antibiotic tolerance, RNA-sequencing, protein chaperones, gene expression

## Abstract

Antibiotic tolerance enables antibiotic-susceptible bacteria to withstand prolonged exposure to high concentrations of antibiotics. Although antibiotic tolerance presents a major challenge for public health, its underlying molecular mechanisms remain unclear. Previously, we have demonstrated that *Campylobacter jejuni* develops tolerance to clinically important antibiotics, including ciprofloxacin and tetracycline. To identify cellular responses associated with antibiotic tolerance, RNA-sequencing was conducted on *C. jejuni* after inducing antibiotic tolerance through exposure to ciprofloxacin or tetracycline. Additionally, knockout mutants were constructed for genes exhibiting significant changes in expression levels during antibiotic tolerance. The genes involved in protein chaperones, bacterial motility, DNA repair system, drug efflux pump, and iron homeostasis were significantly upregulated during antibiotic tolerance. These mutants displayed markedly reduced viability compared to the wild-type strain, indicating the critical role of these cellular responses in sustaining antibiotic tolerance. Notably, the protein chaperone mutants exhibited increased protein aggregation under antibiotic treatment, suggesting that protein chaperones play a critical role in managing protein disaggregation and facilitating survival during antibiotic tolerance. Our findings demonstrate that various cellular defense mechanisms collectively contribute to sustaining antibiotic tolerance in *C. jejuni*, providing novel insights into the molecular mechanisms underlying antibiotic tolerance.

## 1 Introduction

Antibiotic tolerance enables antibiotic-susceptible bacteria to survive antibiotic treatments without acquiring resistance, significantly compromising the effectiveness of antibiotic treatment (Levin-Reisman et al., 2017; Liu et al., 2020). Unlike antibiotic resistance, antibiotic tolerance is a transient state in which bacteria can survive antibiotic exposure without undergoing genetic alterations or acquiring resistance genes (Brauner et al., 2016; Levin-Reisman et al., 2019). Both antibiotic tolerance and persistence are common bacterial strategies to survive under antibiotic treatment, and they are often referred to interchangeably (Brauner et al., 2016; Balaban et al., 2019). However, it is important to note that they represent distinct characteristics (Balaban et al., 2019; Ronneau et al., 2021). Antibiotic persistence occurs in a small subpopulation of persister cells that are transiently tolerant to antibiotics and can resume growth upon removal of antibiotic treatment (Balaban et al., 2019; Ronneau et al., 2021). In contrast, antibiotic tolerance refers to a population-wide temporary state where bacteria can withstand and survive the toxic effects of antibiotics (Brauner et al., 2016; Balaban et al., 2019; Westblade et al., 2020). Additionally, persistence is characterized by physiological dormancy as the lethal effects of antibiotics can be evaded in dormant bacteria with extremely slow metabolic and proliferation rates in response to antibiotics, whereas tolerance does not necessarily involve physiological dormancy (Brauner et al., 2016; Balaban et al., 2019). The distinction between antibiotic persistence and tolerance results in distinct time-kill curve patterns. Antibiotic persistence leads to a unique biphasic pattern, characterized by the survival of a tolerant subpopulation followed by the killing of the majority of non-tolerant bacterial populations. In contrast, tolerance produces a monophasic pattern, demonstrating low levels of bacterial killing over time (Brauner et al., 2016; Balaban et al., 2019). Upon the removal of antibiotic stress, bacteria can be resuscitated to their normal physiological state. Recovery from antibiotic persistence from dormancy requires a relatively long time compared to that from tolerance (Meredith et al., 2015; Levin-Reisman et al., 2019).

To investigate tolerance mechanisms using persister cells, specific procedures are required to selectively isolate and enrich a tolerant subpopulation (Balaban et al., 2004; Gefen and Balaban, 2009; Cañas-Duarte et al., 2014; Sulaiman and Lam, 2020). However, the transient nature and scarcity of persister cells present considerable challenges in researching antibiotic tolerance (Huemer et al., 2020). Furthermore, most studies on tolerance have been constrained to a few hours of antibiotic treatment due to the rapid onset of bacterial death (Santi et al., 2021; Shin et al., 2021), which may not accurately reflect clinical scenarios where pathogens are typically exposed to antibiotics over extended periods, ranging from days to weeks (Aliberti et al., 2010; Wilson et al., 2019). It is also critical to note that tolerance and persistence likely involve different cellular pathways, as they are distinct phenomena (Brauner et al., 2016; Westblade et al., 2020). Persister cells survive antibiotics by entering a physiological state of dormancy and slowing metabolic processes, which may enable them to evade the lethal effects of antibiotics (Bollen et al., 2021).

Furthermore, antibiotic tolerance can facilitate the development of antibiotic resistance as extended survival through tolerance can provide antibiotic-susceptible bacteria with the opportunity to acquire antibiotic resistance under antibiotic treatment (Levin-Reisman et al., 2017; Windels et al., 2019; Liu et al., 2020). In a previous study, we discovered that *Campylobacter jejuni* (C. jejuni) develops tolerance when exposed to high concentrations of clinically important antibiotics, including ciprofloxacin (CIP) and tetracycline (TET) (Park et al., 2022). *C. jejuni* is a leading bacterial cause of gastroenteritis, causing 92 million to 300 million infection cases worldwide per year (Kirk et al., 2015). *Campylobacter* infections are generally self-limiting; however, antimicrobial therapies are required for severe infection cases, especially for the elderly and individuals with compromised immune systems (Balaban et al., 2004; Cañas-Duarte et al., 2014; Sulaiman and Lam, 2020). However, *C. jejuni* is increasingly resistant to clinically important antibiotics, particularly fluoroquinolones (FQs), the most commonly prescribed class of antibiotics for oral treatment of various bacterial infections, including gastroenteritis (Andersson and MacGowan, 2003; Schierenberg et al., 2019).

In our previous study, we demonstrated that high antibiotic concentrations promote the generation of reactive oxygen species (ROS) in *C. jejuni* during antibiotic tolerance, leading to DNA mutations resulting in antibiotic resistance, particularly FQ resistance (Park et al., 2022). Moreover, we have found that antioxidation processes play a critical role in maintaining antibiotic tolerance in *C. jejuni*. Our current understanding of the molecular mechanisms of antibiotic tolerance is highly limited (Meredith et al., 2015). Especially, there is a lack of information on how bacteria can address cellular damage resulting from antibiotic treatment. *C. jejuni* offers a unique and feasible model for studying antibiotic tolerance due to its relatively faster growth compared to other tolerance-developing bacteria, such as *Mycobacterium tuberculosis* (Goossens et al., 2020). Utilizing *C. jejuni*, in this study, we reveal the complex interplay of molecular processes that enable bacterial survival under high antibiotic concentrations through tolerance.

## 2 Materials and methods

### 2.1 Bacterial strains and growth conditions

*C. jejuni* NCTC 11168 was used as wild type (WT) in this study. *C. jejuni* strains were grown microaerobically (5% O_2_, 10% CO_2_, and 85% N_2_) at 42°C on Mueller-Hinton (MH) media (Oxoid, Hampshire, UK). *Escherichia coli* MG1655 (ATCC 700926) was grown at 37°C on Luria-Bertani (LB) media (Difco, MI, United States). For the growth of mutants harboring an antibiotic resistance cassette, the culture media were supplemented with antibiotics, including carbenicillin (100 μg/mL), kanamycin (50 μg/mL), or chloramphenicol (12.5 μg/mL).

### 2.2 Time-kill assay

Overnight cultures of *C. jejuni* grown on MH agar were resuspended in 5 mL of MH broth in a 14-mL round-bottom tube (BD Falcon, MA, United States) to an optical density at 600 nm (OD_600_) of 0.08. The bacterial suspension was then incubated with shaking under microaerobic conditions. After 7 h incubation, antibiotic exposure was initiated by adding 100× minimum inhibitory concentrations (MICs) of CIP (6.3 μg/mL) or TET (3.1 μg/mL) (Supplementary Table S1). TET and CIP were used for testing because they are clinically important antibiotics that *C. jejuni* is known to develop tolerance (Luangtongkum et al., 2009; Schierenberg et al., 2019). Moreover, these antibiotics are commonly used in both human and veterinary medicine, making them relevant choices for studying antibiotic tolerance in this pathogen (European Medicines Agency, 2022; US Food and Drug Administration, 2023). Our previous studies show that *C. jejuni* develops antibiotic tolerance when exposed to concentrations greater than 10× MICs, including 100× MICs (Park et al., 2022). To more effectively differentiate non-tolerant populations in our experiment, we opted to use 100× MICs. Moreover, employing high antibiotic concentrations is a commonly accepted experimental method for inducing antibiotic tolerance (Fridman et al., 2014; Mechler et al., 2015). The concentrations were determined based on the MICs of WT. After 24, 48, and 72 h incubation, 100 μL of *C. jejuni* cultures were harvested and washed with ice-cold phosphate-buffered saline (PBS) three times. After washing, bacterial cells were resuspended in 1 mL of PBS and diluted with MH broth. Five microliters of bacterial cells were spotted onto MH agar and incubated for 2 days to assess viability. To examine the effect of an efflux pump inhibitor on antibiotic tolerance, *C. jejuni* was incubated with phenylalanine-arginine β-naphthylamide (PAβN) (10 or 20 μg/mL) for 7 h in the presence of antibiotics as described above. The assay was also conducted with *E. coli*. *E. coli* MG1655 was incubated in LB broth with shaking under aerobic conditions. At the exponential growth phase, the bacterial population was adjusted to a concentration of 10^8^ CFU/mL. Subsequently, the cultures were treated with 100× MICs of CIP (1.6 μg/mL) or TET (50 μg/mL). The samples were harvested after incubation for 2, 4, 8, 12, 24, and 48 h. After washing with ice-cold PBS three times, the bacterial cells were resuspended in PBS, diluted, and spotted onto the LB agar plates. After 12 h of incubation, bacterial viability was assessed.

### 2.3 Total RNA extraction, RNA-Seq, and analysis

Overnight cultures of *C. jejuni* NCTC 11168 grown on MH agar were harvested and suspended in MH broth to an OD_600_ of 0.08. A 3 mL bacterial suspension in a 19 mL glass culture tube was incubated for 7 h with shaking under microaerobic conditions. After 7 h, cultures were treated with 100× MICs of either CIP (6.3 μg/mL) or TET (3.1 μg/mL) for 24 h. Bacterial cultures (2.5 mL) were treated with 5% ice-cold phenol-ethanol solution, and total bacterial RNAs were isolated using the RNeasy Minikit (Qiagen, Hilden, Germany) according to the manufacturer’s instructions. The quantity and quality of total RNA samples were assessed using a NanoPhotometer N60 (Implen, Munich, Germany), and three biological replicate RNA samples were sent to Macrogen (Seoul, Republic of Korea) for RNA sequencing.

The quality and quantity of total RNA were further evaluated using an Agilent Technologies 2100 Bioanalyzer, ensuring an RNA integrity number (RIN) value greater than 7. A library was prepared independently with 1 μg of total RNA for each sample by Illumina TruSeq Stranded mRNA Sample Prep Kit (Illumina, Inc., CA, United States). Initially, bacterial rRNA-depleted samples were prepared by using the NEBNext rRNA Depletion kit (NEB, NA, United States). After rRNA depletion, the remaining RNA was fragmented into small pieces using divalent cations under elevated temperature. The RNA fragments were converted into first-strand cDNA using SuperScript II reverse transcriptase (Invitrogen, MA, United States) and random primers. This is followed by second-strand cDNA synthesis using DNA Polymerase I, RNase H, and dUTP. These cDNA fragments underwent an end repair process, the addition of a single ‘A’ base, and ligation of the adapters. The products were then purified, enriched with PCR, and processed to create the final cDNA library. Library quantification was carried out using KAPA Library Quantification kits for Illumina Sequencing platforms, and qualification was performed using the TapeStation D1000 ScreenTape (Agilent, CA, United States). Indexed libraries were then submitted to an Illumina NovaSeq 6000 (Illumina, Inc., CA, United States), employing paired-end (2 × 100 bp) sequencing by Macrogen (Seoul, Republic of Korea).

The expression level of each gene was normalized by calculating reads per kilobase per million mapped reads (RPKM) using CLC Workbench. Fold change was determined in comparison to the untreated control (No antibiotics). Differentially expressed genes (DEGs; fold change ≥ 2 or ≤ −2; *p* < 0.05) were filtered and visualized using the Gitools.

### 2.4 Quantitative real-time PCR

Total RNA was extracted as described above, and cDNA was synthesized using cDNA EcoDry premix (Takara Bio Inc., Kusatsu, Japan). qRT-PCR was performed in a 20 μL reaction volume containing cDNA, iQ SYBR Green supermix (Bio-Rad, CA, United States), and each primer, using the CFX Connect real-time PCR detection system (Bio-Rad, CA, United States). The primer sets used in qRT-PCR are described in Supplementary Table S2. The cycling conditions were as follows: 95°C for 5 min; 40 cycles at 95°C for 15 s, 55°C for 15 s, and 72°C for 30 s; followed by 72°C for 7 min. The transcriptional levels of each gene were normalized to the 16S rRNA gene.

### 2.5 Construction of *Campylobacter jejuni* mutants and complemented strains

The *dnaK*, *clpB*, *groESL*, *cheY*, *ruvC*, *cmeC*, and *cmeF* knockout mutants were constructed as described previously (Kim et al., 2023). The *aphA3* (kanamycin resistance) cassette and *cat* (chloramphenicol resistance) cassette were amplified with PCR from pMW10 and pRY112 plasmids, respectively, using primers described in Supplementary Table S2. Flanking regions of the target genes were also amplified by PCR (Supplementary Table S2). Subsequently, the PCR products and pUC19 were digested using BamHI and SalI enzymes, followed by ligation. The resulting plasmids were amplified by inverse PCR and ligated with *aphA3* for constructing *dnaK*, *clpB*, *groESL*, *cheY*, *ruvC*, and *cmeC* mutants or *cat* for the *cmeF* mutant (Supplementary Table S2). The constructed suicide plasmid was electroporated into *C. jejuni*, and the mutations were confirmed with PCR and sequencing. For the construction of *flaA* and *flaB* mutants, natural transformation was performed as previously described (Wang and Taylor, 1990). Briefly, the genomic DNAs were extracted from *C. jejuni* 81-176 Δ*flaA*::*cat* (Hwang et al., 2011) and *C. jejuni* 81-176 Δ*flaB*::*cat* mutants in the laboratory collection, digested by SphI and NdeI for Δ*flaA*, and SphI and SalI for Δ*flaB*, respectively. The DNA was spotted directly on the *C. jejuni* cultures grown overnight on MH agar plates and further incubated for 5 h under microaerobic conditions. The bacterial culture was collected and spread on the MH agar plate containing chloramphenicol (12.5 μg/mL). After incubation for 48 h, the chloramphenicol-resistant colonies were selected, and the mutations were confirmed by PCR and sequencing using specific primer sets (Supplementary Table S2). In addition, complemented strains were constructed using the chromosomal integration method (Kim et al., 2023). The genes (*dnaK*, *clpB*, *groESL*, *ruvC*, *flaA*, *cmeC*, and *cmeF*) were amplified with PCR using primers listed in Supplementary Table S2, and digested by XbaI or NotI and ligated with the pFMBcomCM plasmid (Hwang et al., 2012) (for *dnaK*, *clpB*, *groESL*, *ruvC*, and *cmeC*), or pFMBcomC (Hwang et al., 2011) (for *flaA*, *cmeF*, and *fur*). The complementation plasmids were introduced to the corresponding mutant strains by electroporation. The complementation into the bacterial chromosome was confirmed with PCR and sequencing. The Δ*fur* mutant strain was previously constructed (Kim et al., 2011).

### 2.6 Cross-section transmission electron microscopy

*C. jejuni* cells were treated with antibiotics for 24 h, as described above. After harvest, *C. jejuni* cells were washed with ice-cold PBS three times and fixed with Karnovsky’s fixative solution overnight at 4°C. The pellets were washed with 0.05 M sodium cacodylate buffer three times, following post-fixation with 1% osmium tetroxide in the same buffer at room temperature for 1 h. After washing with distilled water three times, en bloc staining was performed with 0.5% uranyl acetate overnight at 4°C. The next day, the samples were washed with distilled water three times, and dehydrated in a series of ethanol gradients (30, 50, 70, 80, 90, and 100%) for 20 min in each step while slowly rotating. The final 100% ethanol step was repeated three times. Finally, cells were incubated in a 1:1 mixture of Spurr’s resin (Wallis and Griffin, 1973) and ethanol for 90 min at room temperature while slowly rotating and subsequently left in a mixture of 2:1 Spurr’s resin and ethanol at room temperature for 90 min while slowly rotating. The cells were placed in 100% Spurr’s resin and incubated overnight while slowly rotating. The next day, samples were embedded in fresh 100% epoxy resin for 3 h and replaced with fresh 100% epoxy resin. The resin was polymerized for 2 days in an oven at 70°C. The samples cut with an ultramicrotome UC7 (Leica, Wetzlar, Germany) were placed on copper grids and double-stained with 2% uranyl acetate and 3% lead citrate. The sections were observed on a JEM-1010 TEM (JEOL, Tokyo, Japan) operated at 80 kV.

### 2.7 Confocal fluorescence microscopy

*C. jejuni* cells were treated with antibiotics for 24 h, as described above. *C. jejuni* cells were then washed with ice-cold PBS three times. The pellets were resuspended in 1:500 diluted Proteostat dye (Enzo Life Sciences, NY, United States) and incubated for 20 min in the dark at RT. The cells were simultaneously incubated with SYTO9 (Invitrogen, MA, United States) for 15 min. Then, the sample was washed with PBS. The cells were fixed with 4% paraformaldehyde for 30 min at RT. After washing with PBS, the pellets were resuspended with PBS. 5 μL of each sample was placed on the slides. Confocal images of *C. jejuni* cells were captured using a laser scanning confocal microscope SP8X (Leica, Wetzlar, Germany) using a 488 nm argon laser and a 580 nm emission filter. Images were digitally captured and analyzed with LAS X Software (Leica, Wetzlar, Germany).

### 2.8 Measurement of intracellular iron levels

The intracellular iron levels were measured as previously described (Hur et al., 2022). Briefly, *C. jejuni* cells were treated with antibiotics for 24 h, as described above. *C. jejuni* cells were then washed with ice-cold PBS three times. After resuspending the pellet with PBS, the cells were disrupted by sonication. Samples were mixed with an iron detection reagent (6.5 mM ferrozine, 6.5 mM neocuproine, 2.5 M ammonium acetate, and 1 M ascorbic acid) and incubated at RT for 30 min. The intracellular iron level was calculated by comparing it to a standard curve obtained from a 1 mM FeCl_3_ (Sigma-Aldrich, MO, United States) solution. The absorbance was measured at 550 nm using a SpectraMax i3 platform (Molecular Devices, CA, United States). The intracellular iron levels were normalized to total protein concentrations, which were determined by the Bradford assay (Bio-Rad, CA, United States).

### 2.9 Statistical analysis

All assays were conducted using three independent biological replicates. The figures indicate the mean value and error bars represent the standard deviations of each experiment. Student’s *t* test was used to determine significance using GraphPad Prism software v8.0.1. The *p*-value threshold was set at *p < 0.05; **p < 0.01; ***p < 0.001; ****p < 0.0001.

## 3 Results

### 3.1 Transcriptome changes during antibiotic tolerance in *Campylobacter jejuni*

**Figure 1:**
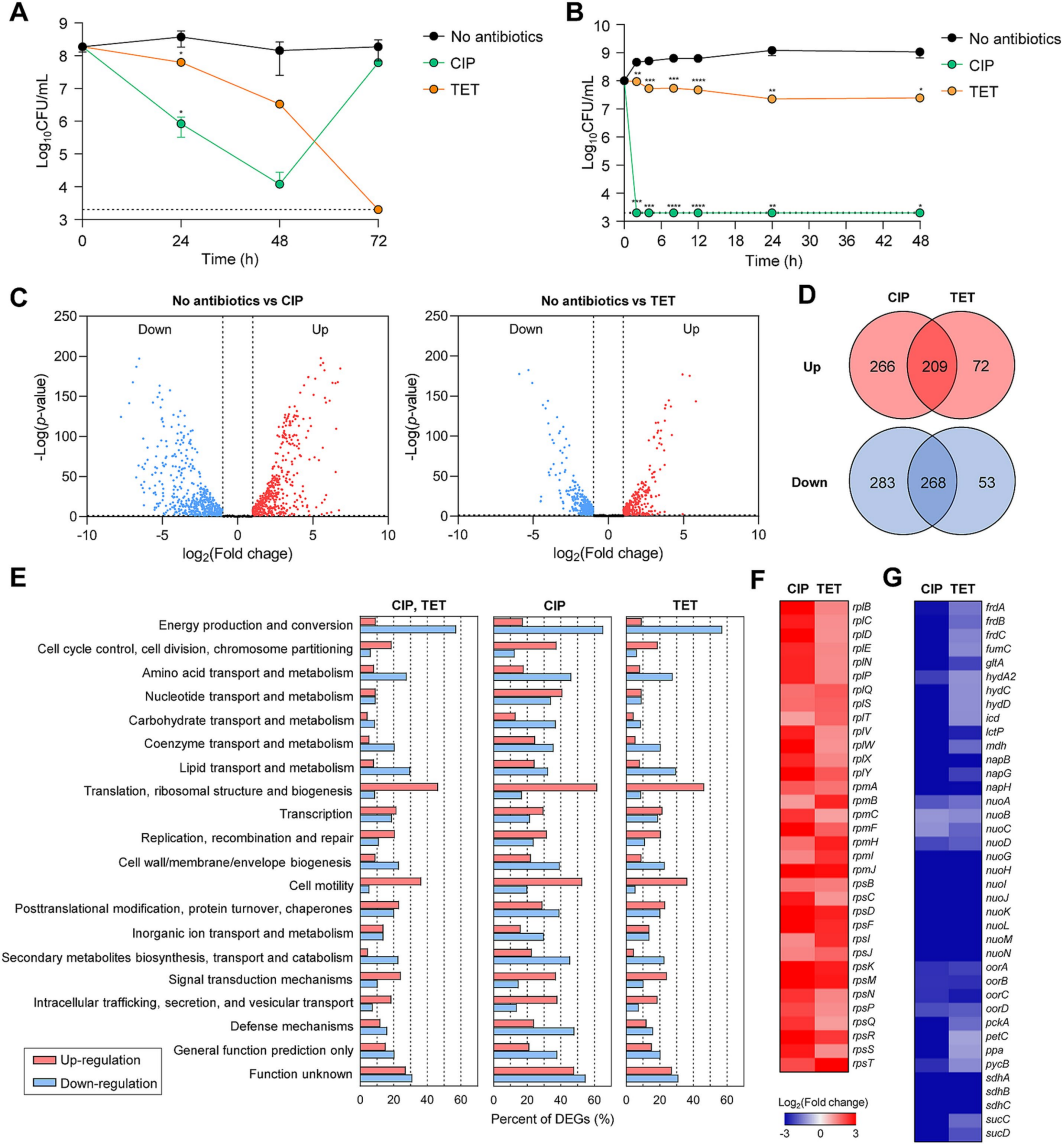
Antibiotic tolerance and transcriptomic changes in *C. jejuni* in the presence of high concentrations of ciprofloxacin (CIP) or tetracycline (TET). **(A,B)** Induction of antibiotic tolerance in *C. jejuni* **(A)** and *E. coli* **(B)** by exposure to 100× minimum inhibitory concentrations (MICs) of CIP (6.3 μg/mL and 1.6 μg/mL, respectively) or TET (3.1 μg/mL and 50 μg/mL, respectively). Dashed line shows detection threshold. Error bars represent the standard deviations of three biological replications. The data were statistically analyzed by the Student’s *t* test in comparison with the untreated control (No antibiotics); *p < 0.05; **p < 0.01; ***p < 0.001; ****p < 0.0001. **(C–G)** The transcriptomic changes in *C. jejuni* after exposure to 100× MICs of ciprofloxacin (CIP; 6.3 μg/mL) or tetracycline (TET; 3.1 μg/mL) based on RNA-Seq. Fold change was defined compared to the untreated control. Volcano plots **(C)** and Venn diagrams **(D)** depicting differentially expressed genes (DEGs) in *C. jejuni* after exposure to 100× MICs of CIP or TET. The percentages of DEGs **(E)** in *C. jejuni* during antibiotic tolerance. Heat maps of the genes associated with translation, ribosomal structure, and biogenesis **(F)** or energy production and conversion **(G)** after exposure to 100× MICs of CIP or TET. The heat maps were constructed with Gitools.

In order to understand transcriptome changes underlying antibiotic tolerance, we performed RNA-Seq after exposing *C. jejuni* to high concentrations of CIP or TET. These two antibiotics were chosen based on our previous findings that *C. jejuni* can survive in the presence of high concentrations of these antibiotics through tolerance for an extended period (Park et al., 2022). Notably, CIP and TET have different modes of action, with CIP disrupting bacterial DNA synthesis by targeting DNA gyrase (Appelbaum and Hunter, 2000) and TET inhibiting protein synthesis by targeting the 30S subunit of ribosomes (Grossman, 2016). Upon exposure to 100× MICs of CIP, *C. jejuni* underwent viability reduction for 2 days, followed by the emergence of CIP-resistant populations (Figure 1A). The survival pattern of *C. jejuni* in the presence of these antibiotics differs from that of *E. coli*. In *E. coli*, CIP significantly reduces viability within a few hours, followed by the survival of persister cells at extremely low levels (Figure 1B). Moreover, high TET concentrations led to the killing of *C. jejuni* (Figure 1A), whereas TET exhibited only bacteriostatic activity in *E. coli* (Figure 1B).

The results of the RNA-Seq analysis have revealed that significant transcriptome changes occur during antibiotic tolerance. Compared to the untreated sample, CIP was found to induce differential expression in a total of 1,026 genes, including 475 upregulated genes and 551 downregulated genes (Figures 1C,D), whereas TET exhibited differential expression in a total of 602 genes, with 281 upregulated genes and 321 downregulated genes (Figures 1C,D). Additionally, a total of 477 genes were differentially expressed in both CIP and TET (Figure 1D). Notably, CIP upregulated genes involved in various cellular functions, such as cell cycle control, cell division, chromosome partitioning, and cell motility, while downregulating genes associated with amino acid transport and metabolism, secondary metabolites biosynthesis, and defense mechanisms (Figure 1E). On the other hand, both CIP and TET commonly increased the transcription of genes associated with translation and ribosomal structure, while downregulating those related to energy production and conversion (Figures 1E,G). Notably, genes involved in protein translation and tRNA genes were upregulated (Figure 1F), indicating that *C. jejuni* is not dormant during antibiotic tolerance and actively synthesizes proteins to respond to antibiotic stress.

### 3.2 Protein chaperones contribute to sustaining antibiotic tolerance

**Figure 2:**
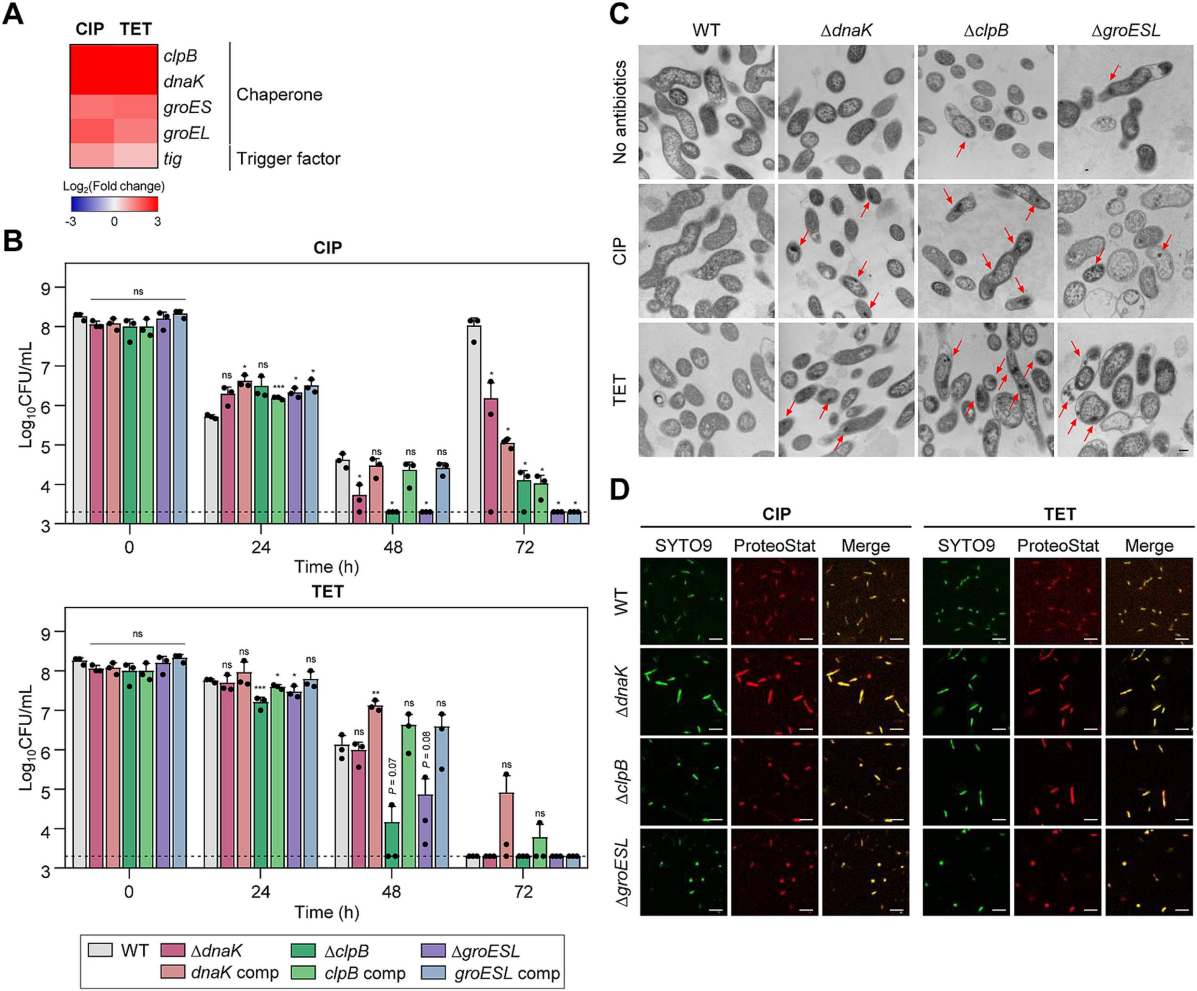
Effects of chaperones on antibiotic tolerance in *C. jejuni* in the presence of high concentrations of ciprofloxacin (CIP) or tetracycline (TET). **(A)** Heat maps of the genes associated with chaperones after exposure to 100× minimum inhibitory concentrations (MICs) of CIP (6.3 μg/mL) or TET (3.1 μg/mL). The heat maps were constructed with Gitools. **(B)** Induction of antibiotic tolerance by exposure to 100× MICs of CIP (6.3 μg/mL) or TET (3.1 μg/mL). The concentrations were determined based on the MICs of WT. Dashed line shows detection threshold. Error bars represent the standard deviations of three biological replications. The data were statistically analyzed by the Student’s *t* test in comparison with WT. *p < 0.05; **p < 0.01; ***p < 0.001; ns, not significant; WT, wild type; Δ*dnaK*, *dnaK* mutant; *dnaK* comp, *dnaK*-complemented strain; Δ*clpB*, *clpB* mutant; *clpB* comp, *clpB*-complemented strain; Δ*groESL*, *groESL* mutant; *groESL* comp, *groESL*-complemented strain. **(C)** Formation of protein aggregates induced after exposure to 100× MICs of CIP or TET. The concentrations were determined based on the MICs of WT. Red arrows indicated protein aggregates observed by cross-section transmission electron microscopy (TEM). The formation of protein aggregates was compared to the untreated control (No antibiotics). The scale bar represents 0.2 μm. **(D)** Protein aggregates were visualized with fluorescent probes. Live cells were stained with SYTO9 (green) and protein aggregates with Proteostat (red). The merged images are shown in yellow. The scale bar represents 5 μm.

Protein chaperones are critical for assisting in proper protein folding and disaggregation, particularly under stress conditions (Mayer, 2021). The three major bacterial chaperone complexes are the trigger factor, the DnaK-DnaJ, and the GroEL-GroES complexes (Sabate et al., 2010). RNA-Seq analysis has revealed that genes encoding these chaperone complexes, specifically *clpB*, *dnaK*, *groES*, *groEL*, and *tig*, are significantly upregulated during antibiotic tolerance (Figure 2A), suggesting that these chaperones play an important role in bacterial survival under antibiotic treatment. The Δ*dnaK*, Δ*clpB*, and Δ*groESL* mutant strains were constructed to further examine the role of protein chaperones in conferring antibiotic tolerance. The deletion mutants exhibited no substantial growth impairments compared to WT in the absence of antibiotic exposure (Supplementary Figure S1A). Notably, Δ*dnaK*, Δ*clpB*, and Δ*groESL* mutations have been shown to compromise antibiotic tolerance, resulting in significant viability reductions after 48 h of antibiotic exposure (Figure 2B). This was particularly evident under treatment with TET, where Δ*clpB* and Δ*groESL* mutations facilitated bacterial cell death (Figure 2B).

Environmental stress can disrupt protein homeostasis, leading to the formation of insoluble protein aggregates within bacterial cells (Vaubourgeix et al., 2015; Schramm et al., 2019). TEM has been employed to investigate whether antibiotic exposure induces protein aggregation during antibiotic tolerance. Compared to WT, more protein aggregates were detected in Δ*dnaK*, Δ*clpB*, and Δ*groESL* mutants (Figure 2C). Additionally, the use of Proteostat, a fluorescent dye that selectively binds to misfolded and aggregated proteins (Bagnoli et al., 2023), has corroborated these observations. Consistently, enhanced protein aggregation was observed in the chaperone mutants compared to WT (Figure 2D). In contrast, there was no discernible difference in the negative controls without antibiotic treatment (Supplementary Figure S2). These findings indicate the critical function of chaperone proteins in bacterial survival during antibiotic tolerance.

### 3.3 Association of bacterial motility with antibiotic tolerance

**Figure 3:**
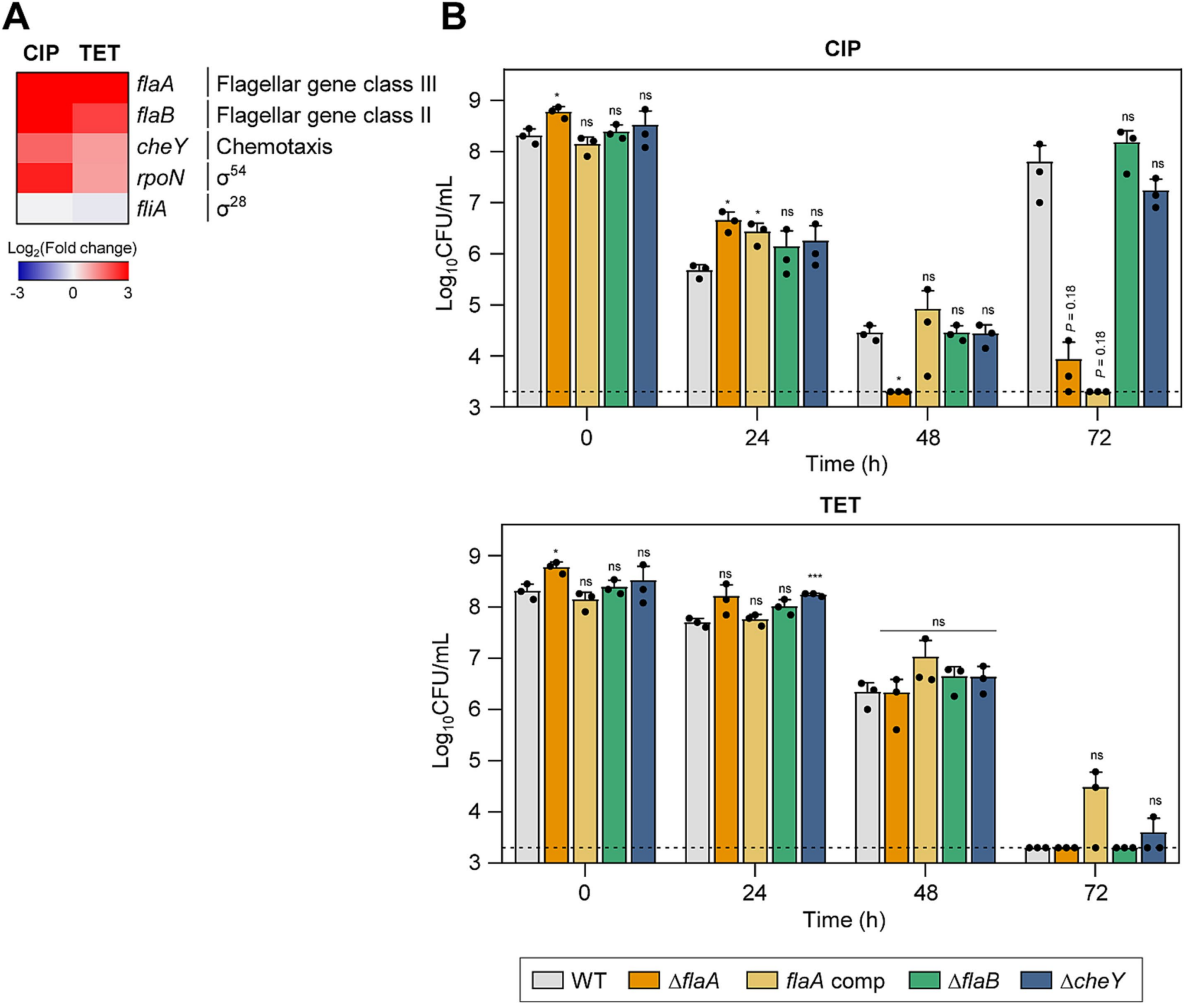
Effects of motility-related genes on antibiotic tolerance in *C. jejuni* in the presence of high concentrations of ciprofloxacin (CIP) or tetracycline (TET). **(A)** Heat maps of the genes associated with motility after exposure to 100× minimum inhibitory concentrations (MICs) of CIP (6.3 μg/mL) or TET (3.1 μg/mL). The heat maps were constructed with Gitools. **(B)** Induction of antibiotic tolerance by exposure to 100× MICs of CIP (6.3 μg/mL) or TET (3.1 μg/mL). The concentrations were determined based on the MICs of WT. Dashed line shows detection threshold. Error bars represent the standard deviations of three biological replications. The data were statistically analyzed by the Student’s *t* test in comparison with WT; *p < 0.05; ***p < 0.001; ns, not significant; WT, wild type; Δ*flaA*, *flaA* mutant; *flaA* comp, *flaA*-complemented strain; Δ*flaB*, *flaB* mutant; Δ*cheY*, *cheY* mutant.

The motility of *C. jejuni* is facilitated by polar flagella that consist of flagellins encoded by *flaA* and *flaB*, which are regulated by the sigma factors FliA and RpoN, respectively (Guerry et al., 1991; Alm et al., 1993). Among these flagellin genes, *flaA* is the major flagellin as its inactivation leads to a loss of motility and a decrease in virulence, whereas an inactivation of *flaB* results in the formation of truncated flagella but does not reduce motility (Alm et al., 1993). Genes related to motility were significantly upregulated during tolerance (Figure 3A), suggesting that bacterial motility is critical for antibiotic tolerance. Additionally, the *cheY* gene, which is integral to bacterial chemotaxis (Yao et al., 1997), was also upregulated during antibiotic tolerance (Figure 3A).

To better understand the contributions of motility and chemotaxis to antibiotic tolerance, we constructed Δ*flaA*, Δ*flaB*, and Δ*cheY* mutants. These mutant strains did not display any significant growth defects compared to WT without antibiotics treatment (Supplementary Figure S1B). When treated with CIP, the Δ*flaA* mutant demonstrated a significant reduction in viability compared to WT, suggesting that *flaA*-mediated motility is crucial for CIP tolerance (Figure 3B). Under TET treatment, there was no observable difference in viability reduction between the mutants and WT (Figure 3B), indicating that the role of motility and chemotaxis in antibiotic tolerance may vary depending on the antibiotic.

### 3.4 DNA repair is critical for antibiotic tolerance

**Figure 4:**
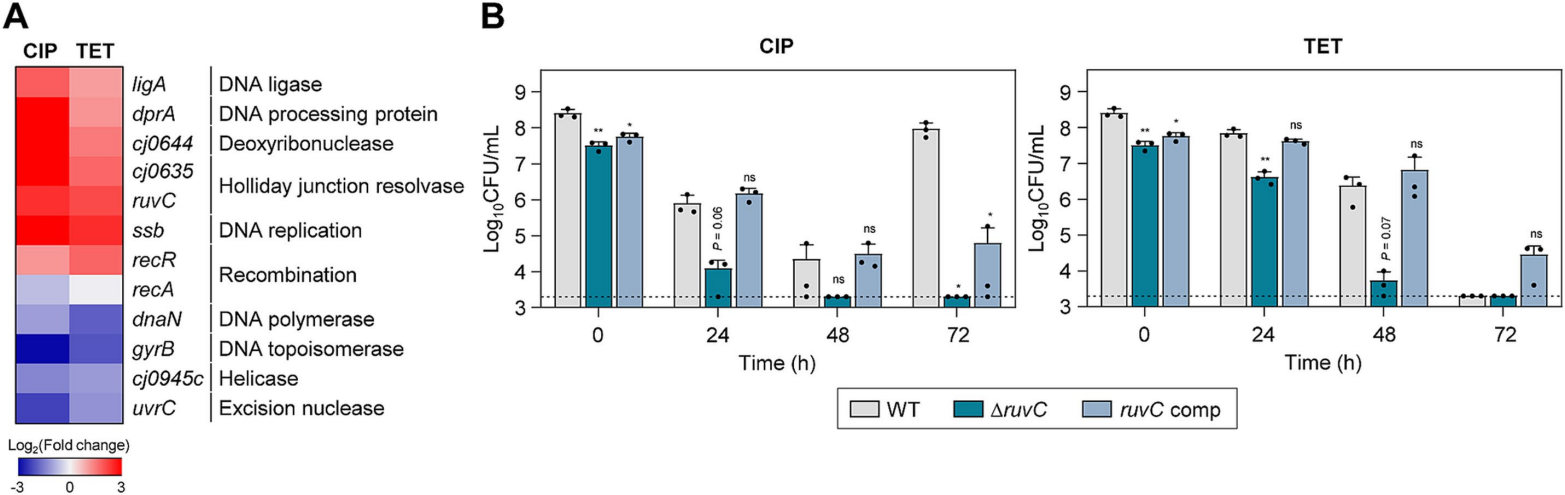
Effects of DNA repair genes on antibiotic tolerance in *C. jejuni* in the presence of high concentrations of ciprofloxacin (CIP) or tetracycline (TET). **(A)** Heat maps of the genes associated with DNA repair after exposure to 100× minimum inhibitory concentrations (MICs) of CIP (6.3 μg/mL) or TET (3.1 μg/mL). The heat maps were constructed with Gitools. **(B)** Induction of antibiotic tolerance by exposure to 100× MICs of CIP (6.3 μg/mL) or TET (3.1 μg/mL). The concentrations were determined based on the MICs of WT. Dashed line shows detection threshold. Error bars represent the standard deviations of three biological replications. The data were statistically analyzed by the Student’s *t* test in comparison with WT; *p < 0.05; **p < 0.01; ns, not significant; WT, wild type; Δ*ruvC*, *ruvC* mutant; *ruvC* comp, *ruvC*-complemented strain.

A number of genes involved in DNA repair were significantly upregulated during antibiotic tolerance (Figure 4A). Specifically, the *ssb* gene, which encodes the single-stranded DNA-binding protein crucial for DNA replication, recombination, and repair (Xu et al., 2023), was upregulated by exposure to high concentrations of antibiotics. Additionally, antibiotic treatment also upregulated *dprA* encoding DNA processing protein A (DprA), which assists in the integration of single-stranded DNA into the genome and is involved in natural transformation in *C. jejuni* (Takata et al., 2005). Interestingly, antibiotic treatment did not upregulate *recA* (Figure 4A), presumably due to the absence of SOS response systems in *C. jejuni* (Parkhill et al., 2000).

To evaluate the impact of DNA repair systems on antibiotic tolerance, we constructed a Δ*ruvC* mutant and measured its viability in the presence of 100× MICs of CIP or TET. In the absence of antibiotic exposure, the deletion of the *ruvC* gene did not lead to a significant growth defect compared to WT (Supplementary Figure S1C). RuvC plays an important role in DNA repair and recombination by cleaving Holliday junctions (Iwasaki et al., 1991). The *ruvC* was selected for testing because its transcriptional level was significantly enhanced by both CIP and TET (Figure 4A). Moreover, the Δ*ruvC* mutation severely impaired antibiotic tolerance in *C. jejuni* (Figure 4B). Additionally, the Δ*ruvC* mutation sensitized *C. jejuni* to antibiotics, displaying a decrease in the MICs of CIP and TET by 4-fold and 2-fold, respectively (Supplementary Table S1).

It is noteworthy that the upregulation of DNA repair genes was also observed during tolerance induced by TET, a broad-spectrum antibiotic that works by inhibiting protein synthesis in bacteria (Figure 4A). Potentially, TET exposure triggers a general stress response, which could include the activation of various protective mechanisms, including DNA repair systems. Alternatively, while TET does not directly target DNA, its effects on protein synthesis might indirectly lead to DNA stress or damage. Furthermore, the inactivation of *ruvC* markedly diminished bacterial viability in the presence of TET (Figure 4B). These findings indicate the importance of DNA repair processes in maintaining antibiotic tolerance, regardless of the mode of action of an antibiotic used for tolerance induction.

### 3.5 Drug efflux pumps contribute to maintaining antibiotic tolerance

**Figure 5:**
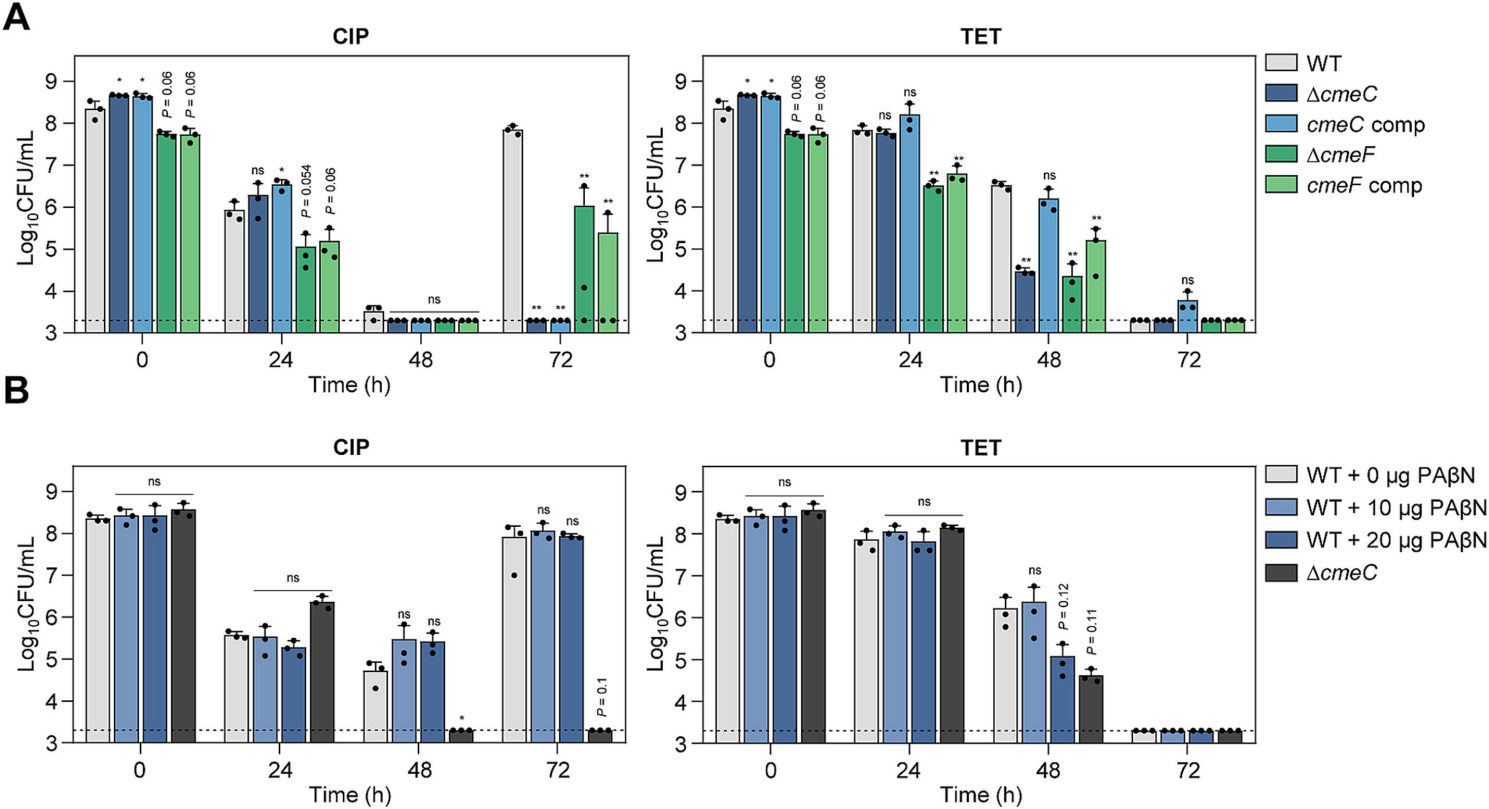
Effects of drug efflux pumps on antibiotic tolerance in *C. jejuni* in the presence of high concentrations of ciprofloxacin (CIP) or tetracycline (TET). **(A,B)** Induction of antibiotic tolerance in efflux pump knockout mutants **(A)** by exposure to 100× minimum inhibitory concentrations (MICs) of CIP (6.3 μg/mL) or TET (3.1 μg/mL). The concentrations were determined based on the MICs of WT. Induction of antibiotic tolerance in WT in the presence of phenylalanine-arginine β-naphthylamide (PAβN), an efflux pump inhibitor **(B)** after exposure to 100× MICs of CIP (6.3 μg/mL) or TET (3.1 μg/mL). Dashed line shows detection threshold. Error bars represent the standard deviations of three biological replications. The data were statistically analyzed by the Student’s *t* test in comparison with WT; *p < 0.05; **p < 0.01; ns, not significant; WT, wild type; Δ*cmeC*, *cmeC* mutant; *cmeC* comp, *cmeC*-complemented strain; Δ*cmeF*, *cmeF* mutant; *cmeF* comp, *cmeF*-complemented strain.

Drug efflux pumps play a critical role in antibiotic resistance by reducing the intracellular concentration of antibiotics (Du et al., 2018; Colclough et al., 2020; Kumawat et al., 2023). In *C. jejuni*, CmeABC is the primary efflux system that confers resistance across various antibiotic classes (Lin et al., 2002; Wieczorek and Osek, 2013; Sharifi et al., 2021). CmeDEF is another drug efflux pump that operates alongside CmeABC to maintain cell viability under antibiotic treatment (Akiba et al., 2006). Transcriptomic analysis showed that the expression of these efflux pump genes is modulated in response to antibiotic exposure (Supplementary Figure S3). To further elucidate the role of these pumps during antibiotic tolerance, we constructed Δ*cmeC* and Δ*cmeF* mutants. CmeABC and CmeDEF are the resistance-nodulation-cell division (RND)-type efflux pumps, which are composed of three proteins spanning the cytoplasmic space and both cell membranes; thus, the absence of any one component renders the entire pump nonfunctional. Without antibiotic treatment, the mutants showed comparable growth to WT (Supplementary Figure S1D). Under CIP treatment, the viability reductions in these efflux pump mutants were slightly more substantial compared to WT after 48 h (Figure 5A). In contrast to WT, notably, the emergence of FQ-resistant strains was not observed in the Δ*cmeC* mutant after 72 h (Figure 5A). In the presence of 100× MICs of TET, tolerance was significantly compromised in both Δ*cmeC* and Δ*cmeF* mutants (Figure 5A).

The role of efflux pumps in antibiotic tolerance was also assessed using the efflux pump inhibitor PAβN. Similarly, PAβN did not affect bacterial viability during CIP-induced tolerance, whereas PAβN significantly reduced viability in the presence of 100× MICs of TET (Figure 5B; Supplementary Figure S1E). These results suggest that drug efflux pumps may contribute to antibiotic tolerance depending on the antibiotic.

### 3.6 Increased iron accumulation during antibiotic tolerance

**Figure 6:**
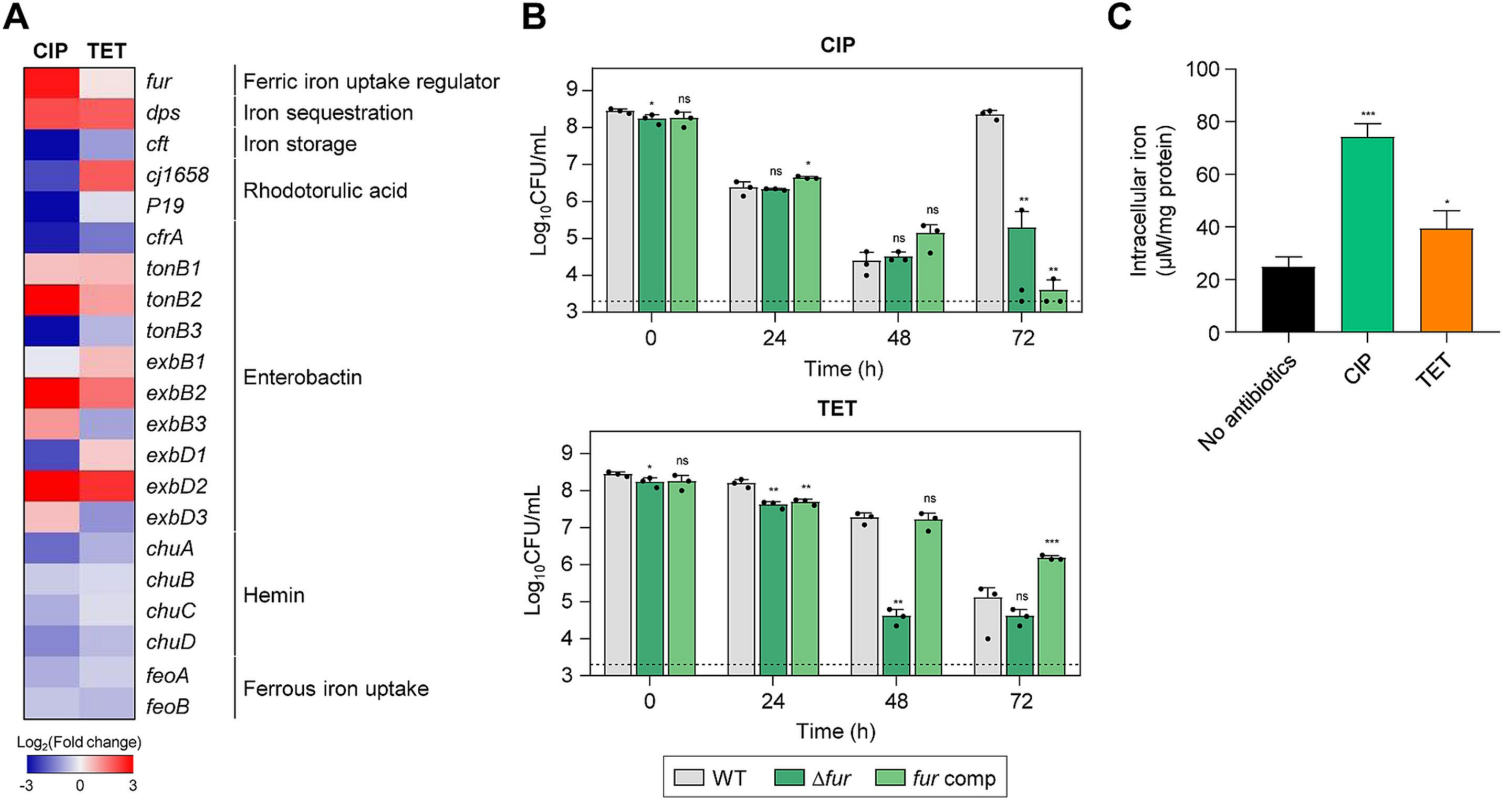
Effects of iron accumulation in *C. jejuni* in the presence of high concentrations of ciprofloxacin (CIP) or tetracycline (TET). **(A)** Heat maps of the genes associated with iron metabolism after exposure to 100× minimum inhibitory concentrations (MICs) of CIP (6.3 μg/mL) or TET (3.1 μg/mL). The heat maps were constructed with Gitools. **(B)** Induction of antibiotic tolerance by exposure to 100× MICs of CIP (6.3 μg/mL) or TET (3.1 μg/mL). The concentrations were determined based on the MICs of WT. Dashed line shows detection threshold. Error bars represent the standard deviations of three biological replications. The data were statistically analyzed by the Student’s *t* test in comparison with WT; *p < 0.05; **p < 0.01; ***p < 0.001; ns, not significant; WT, wild type; Δ*fur*, *fur* mutant; *fur* comp, *fur*-complemented strain. **(C)** The intracellular iron content in *C. jejuni* after exposure to 100× MICs of CIP (6.3 μg/mL) or TET (3.1 μg/mL). Error bars represent the standard deviations of three biological replications. The data were statistically analyzed by the Student’s *t* test in comparison with the untreated control (No antibiotics); *p < 0.05; ***p < 0.001.

RNA-Seq analysis has also revealed a notable upregulation of *fur* transcription during antibiotic tolerance, particularly when tolerance was induced by CIP (Figure 6A). A Δ*fur* mutation significantly compromised the viability in the presence of 100× MICs of TET and reduced the emergence of FQ resistance under the treatment with 100× MICs of CIP (Figure 6B; Supplementary Figure S1F). Interestingly, the level of intracellular iron is significantly increased during antibiotic tolerance (Figure 6C). Iron is a cofactor of a range of proteins essential for fundamental physiological processes (Andrews et al., 2003; Frawley and Fang, 2014). Most Gram-negative bacteria, including *C. jejuni*, maintain cytoplasmic iron levels using Fur, a transcriptional repressor (Lee and Helmann, 2007; Butcher et al., 2012). Iron exists in either the reduced ferrous form (Fe^2+^) or the oxidized ferric form (Fe^3+^). Fe^2+^ can passively diffuse through the outer-membrane porins and is imported by FeoB, which is the only Fe^2+^ transport system that has been identified in *C. jejuni* (Naikare et al., 2006). Fe^3+^ is imported through specific ligand-gated outer-membrane receptor proteins using siderophores, including Fe^3+^-enterochelin (CeuBCDE, CfrA) and Fe^3+^-rhodotorulic acid (P19, Cj1658-63) (Palyada et al., 2004; Miller et al., 2008), During antibiotic tolerance, genes for Fe^3+^-uptake systems involving rhodotorulic acid and hemin, and the Fe^2+^-uptake FeoB were also down-regulated (Figure 6A). Presumably, *fur* transcription was increased so that Fur can prevent further iron uptake to maintain iron homeostasis and reduce cellular toxicity. After the acquisition, iron can be stored in the form of ferritin or incorporated into iron–sulfur complexes (Carrondo, 2003; Krewulak and Vogel, 2008). Moreover, the transcription of *dps*, involved in the sequestration of intracellular free iron (Ishikawa et al., 2003), is significantly increased during tolerance, which is aligned with cellular changes to mitigate cellular toxicity caused by increases in iron levels. These findings suggest that during antibiotic tolerance, *C. jejuni* actively modulates its iron acquisition and storage systems to mitigate the deleterious effects of iron overload. The coordinated regulation of iron uptake and storage genes reflects *C. jejuni*’s adaptive response to maintain iron homeostasis during antibiotic tolerance.

## 4 Discussion

The extensive transcriptomic analysis presented in this study provides new insights into the multifaceted cellular responses during antibiotic tolerance in *C. jejuni*, a leading cause of bacterial gastroenteritis worldwide. The research reveals that *C. jejuni* employs diverse cellular defense mechanisms to withstand high concentrations of clinically important antibiotics. Notably, we discovered that the function of protein chaperones is critical for antibiotic tolerance. In the presence of environmental stress, bacteria may encounter protein aggregation, disrupting protein homeostasis and resulting in the formation of insoluble protein aggregates (Vaubourgeix et al., 2015; Schramm et al., 2019). While protein aggregation is typically linked to adverse cellular effects, such as impaired functions and cell death, it is also considered a survival strategy against antibiotic treatment by inducing bacterial dormancy in persister cells (Bollen et al., 2021). During antibiotic persistence, DnaK and ClpB play a role in maintaining a dormant state, enabling persister cells to survive antibiotic challenges and subsequently return to active growth upon removal of antibiotic stress (Pu et al., 2019). DnaK is a chaperone protein that recognizes and binds to exposed hydrophobic regions on partially misfolded proteins (Calloni et al., 2012; Anglès et al., 2017). It then transfers these partially folded proteins to ClpB, an AAA+ ATPase chaperone, which disaggregates and solubilizes aggregated proteins (Alam et al., 2021). ClpB and DnaK interplay to address protein misfolding and aggregation issues, preventing the formation of toxic protein aggregates in response to stress conditions, including antibiotic treatment (Acebrón et al., 2009; Mogk et al., 2015). While the function of chaperone proteins in bacterial dormancy has been documented in persister cells (Pu et al., 2019), our findings suggest that chaperone proteins facilitate protein disaggregation to maintain bacterial viability during antibiotic tolerance, as antibiotic tolerance does not necessarily involve dormancy (Brauner et al., 2016; Balaban et al., 2019). Although the precise role of molecular chaperones during antibiotic tolerance remains unexplained, our data suggest that protein chaperones contribute to bacterial survival under antibiotic treatment by contributing to protein disaggregation.

We conducted transcriptome analysis after antibiotic exposure for 24 h to capture the effects of extended antibiotic exposure on gene expression. Notably, the transcriptional levels of chaperon genes were increased over the duration of antibiotic exposure from 2 h to 24 h (Supplementary Figure S4), confirming the critical function of chaperons in antibiotic tolerance. Moreover, chaperone mutations did not reduce viability at 24 h but rendered the mutants significantly vulnerable to antibiotics after 48 h. The observed delay in viability reduction of the chaperone mutants may be attributed to the gradual accumulation of cellular damage and stress over time. The absence of chaperones may not immediately result in cell death; instead, misfolded proteins and cellular damage gradually accumulate over time. This cumulative effect may reach a critical threshold after 48 h, leading to a significant decrease in viability. Nevertheless, these findings underscore the significance of protein chaperones in antibiotic tolerance and bacterial survival during prolonged antibiotic exposure.

Our results also further demonstrate the pivotal role of DNA repair in sustaining antibiotic tolerance. This finding strongly suggests that *C. jejuni* is not dormant during tolerance but rather actively engaged in cellular processes to ensure survival. By increasing the expression of DNA repair mechanisms, *C. jejuni* actively works to preserve its genetic integrity, which is essential for maintaining vital physiological functions. This active state in antibiotic tolerance contrasts with the concept of antibiotic persistence, where bacteria often enter a dormant state to evade antibiotic effects (Brauner et al., 2016; Balaban et al., 2019). Additionally, it is noteworthy that *C. jejuni* does not have the SOS response system which is critical for addressing DNA damage in various bacteria (Parkhill et al., 2000). While other bacteria primarily rely on the SOS response-mediated DNA repair system for antibiotic tolerance, *C. jejuni* appears to utilize alternative DNA repair systems that operate independently of the SOS response (Theodore et al., 2013; Podlesek and Žgur Bertok, 2020; Kamat and Badrinarayanan, 2023). During antibiotic tolerance, high antibiotic concentrations lead to the generation of toxic ROS, particularly hydroxyl radicals, which can damage DNA (Park et al., 2022). Presumably, DNA repair during tolerance may contribute to addressing DNA damage and mutations, especially those that interrupt the function of essential genes, thereby ensuring the maintenance of bacterial survival.

Notably, this study has revealed an increase in intracellular iron concentration during antibiotic tolerance (Figure 6C). Although the exact mechanisms are not fully elucidated, potentially, it is known that intracellular free iron can interact with hydrogen peroxide to generate DNA-damaging hydroxyl radicals through the Fenton reaction (Imlay, 2008). Our previous research has indicated a significant elevation of oxidative stress in *C. jejuni* during tolerance, with antioxidant enzymes, such as alkyl hydroperoxide reductase (AhpC), playing a crucial role in maintaining antibiotic tolerance (Park et al., 2022). To mitigate oxidative stress associated with increased intracellular iron levels during antibiotic tolerance, *C. jejuni* should inhibit further iron uptake and sequester intracellular free iron. This is supported by the observed substantial increase in the transcriptional levels of *fur* (a Fe^3+^ uptake repressor) and *dps* (an intracellular iron sequestration protein), along with the down-regulation of *feoB*, which encodes a ferrous iron transporter (Figure 6A). Given the pivotal role of iron in the generation of hydroxyl radicals, maintaining iron homeostasis is crucial for sustaining antibiotic tolerance, likely through the control of oxidative stress.

**Figure 7:**
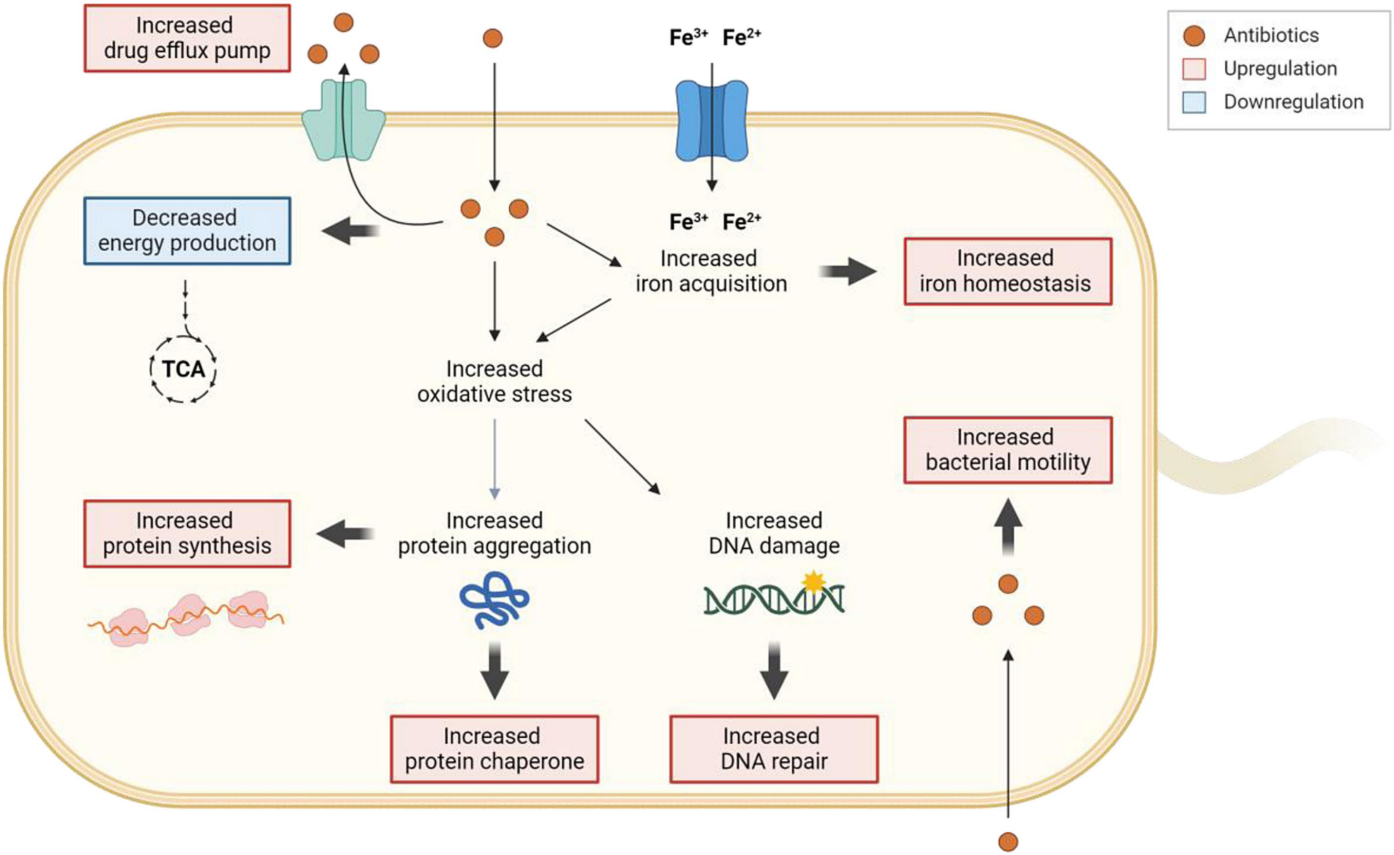
Schematic representation of the cellular responses involved in the development of antibiotic tolerance in *C. jejuni*. When exposed to high levels of antibiotics, drug efflux pumps actively work to decrease the intracellular concentrations of antibiotics. Exposure to high concentrations of antibiotics leads to an accumulation of iron within the cell, subsequently influencing the gene expression of iron acquisition and storage systems to maintain iron homeostasis. The increased oxidative stress caused by antibiotics induces DNA damage, prompting the upregulation of the DNA repair system. Furthermore, oxidative stress may also trigger protein aggregation, resulting in the upregulation of protein synthesis and protein chaperones to disaggregate protein aggregates. Intriguingly, intracellular antibiotics may also stimulate bacterial motility. Additionally, exposure to high concentrations of antibiotics leads to the downregulation of genes associated with energy production. The transcriptional changes are visually represented with bold arrows, with upregulation highlighted in red and downregulation in blue. The figure was created with Biorender.com.

In conclusion, this study has uncovered the diverse cellular responses that contribute to *C. jejuni*’s tolerance to antibiotics. Our findings demonstrate that *C. jejuni* extensively utilizes various cellular defense mechanisms, including antioxidation, protein chaperoning, DNA repair, drug efflux, and iron homeostasis, to survive antibiotic treatment (Figure 7). Particularly, the regulation of intracellular iron levels through iron homeostasis appears to be linked to antibiotic tolerance. These findings have the potential to inform the development of novel strategies to combat antibiotic tolerance and resistance, thereby assisting in tackling this significant issue in global public health.

## Data Availability

The datasets presented in this study can be found in online repositories. The names of the repository/repositories and accession number(s) can be found in the article/Supplementary material.
