# Supplementary material for: Pleiotropic cellular responses underlying antibiotic tolerance in Campylobacter jejuni
Source: Front Microbiol. 2024 Nov 22;15:1493849. doi: 10.3389/fmicb.2024.1493849 (PMC11622253; doi:10.3389/fmicb.2024.1493849)
Supplement: Supplementary file 1 [file Data_Sheet_1.docx]

Supplementary Material

**
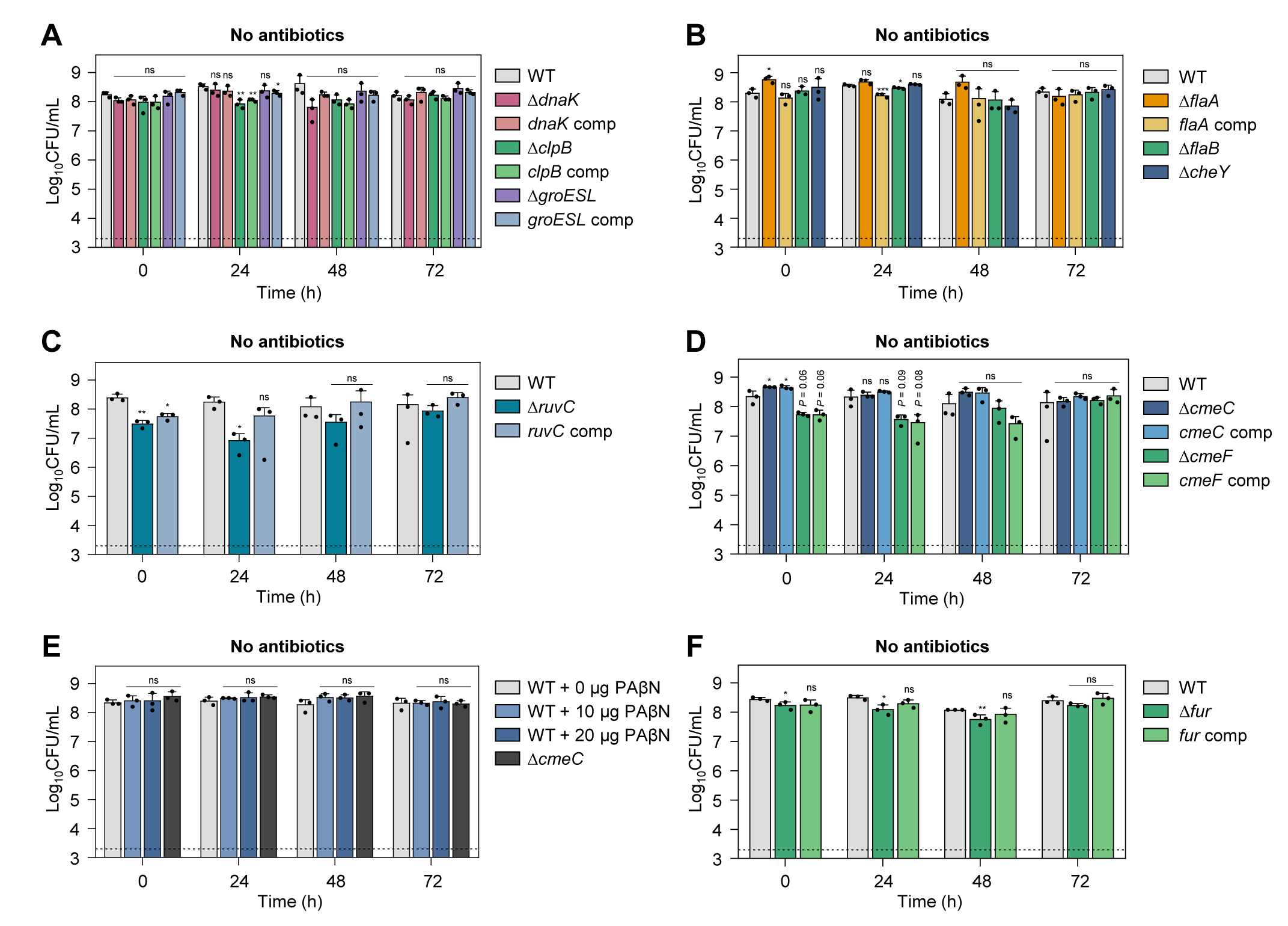
**

**Supplementary Figure 1.** Bacterial survival of the untreated control (No antibiotics) in time-kill assays. **(A-F)** Dashed line shows detection threshold. Error bars represent the standard deviations of three biological replications. The data were statistically analyzed by the Student’s *t* test in comparison with WT; *, *P* < 0.05; **, *P* < 0.01; ns, not significant; WT, wild type; *dnaK* comp, *dnaK*-complemented strain; *clpB* comp, *clpB*-complemented strain; *groESL* comp, *groESL*-complemented strain; *flaA* comp, *flaA*-complemented strain; *ruvC* comp, *ruvC*-complemented strain; *cmeC* comp, *cmeC*-complemented strain; *cmeF* comp, *cmeF*-complemented strain; *fur* comp, *fur*-complemented strain; PAβN, phenylalanine-arginine β-naphthylamide.


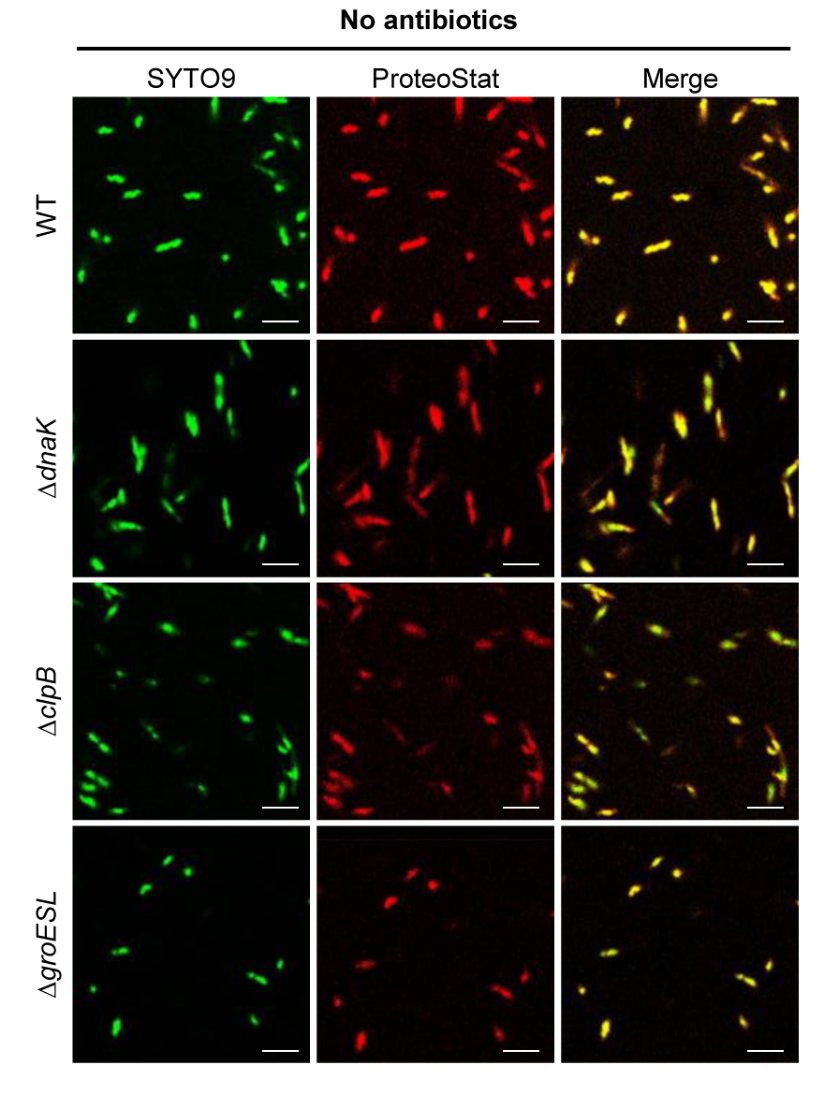


**Supplementary Figure 2.** Confocal fluorescence microscopic images of the untreated control (No antibiotics). Live cells were stained with SYTO9 (green), and protein aggregates with Proteostat® reagent (red). The merged images are shown in yellow. The scale bar represents 5 μm. WT, wild type; Δ*dnaK*, *dnaK* mutant; Δ*clpB*, *clpB* mutant; Δ*groESL*, *groESL* mutant.

**
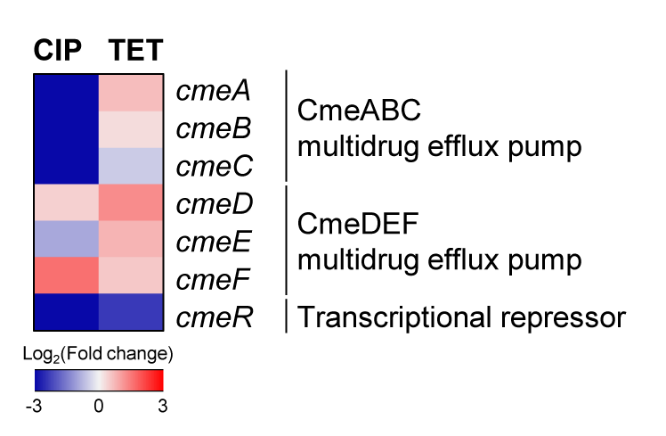
**

**Supplementary Figure 3.** Heat maps of the transcriptional levels of drug efflux pump genes after exposure to 100x MICs of ciprofloxacin (CIP; 6.3 μg/ml) or tetracycline (TET; 3.1 μg/ml). The heat maps were constructed with Gitools.

**
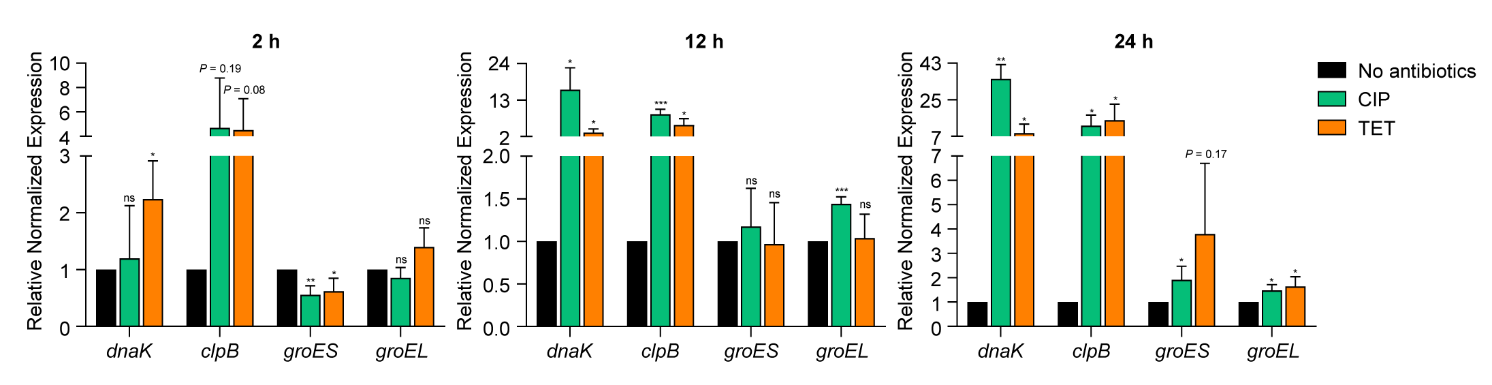
**

**Supplementary Figure 4.** The transcriptional levels of genes associated with chaperones after exposure to 100x minimum inhibitory concentrations (MICs) of ciprofloxacin (CIP; 6.3 μg/ml) or tetracycline (TET; 3.1 μg/ml). At each time point (2, 12, and 24 h), RNA was extracted and analyzed by qRT-PCR. The expression values were normalized with the untreated control (No antibiotics). Error bars represent the standard deviations of three biological replications. The data were statistically analyzed by Student’s *t* test in comparison with the untreated control (No antibiotics); *, *P* < 0.05; **, *P* < 0.01; ***, *P* < 0.001; ns, not significant.

**Supplementary Table 1. The minimum inhibition concentrations (MICs) of *Campylobacter* strains.**

| **Strain**^1^ | **MIC (μg/mL)** | |
| --- | --- | --- |
|  | **Ciprofloxacin**^2^ | **Tetracycline** |
| WT | 0.063 | 0.031 |
| *∆dnaK* | 0.063 | 0.031 |
| *dnaK* comp | 0.063 | 0.031 |
| *∆clpB* | 0.063 | 0.031 |
| *clpB* comp | 0.063 | 0.031 |
| *∆groESL* | 0.063 | 0.031 |
| *groESL* comp | 0.125 | 0.031 |
| *∆flaA* | 0.063 | 0.031 |
| *flaA* comp | 0.063 | 0.031 |
| *∆flaB* | 0.063 | 0.031 |
| *∆cheY* | 0.063 | 0.031 |
| *∆ruvC* | **0.015** | 0.015 |
| *ruvC* comp | 0.063 | 0.031 |
| *∆cmeC* | **0.015** | 0.015 |
| *cmeC* comp | 0.031 | 0.015 |
| *∆cmeF* | 0.063 | 0.031 |
| *cmeF* comp | 0.063 | 0.031 |
| *∆fur* | 0.063 | 0.063 |
| *fur* comp | 0.063 | 0.031 |

^1^WT, wild type; Δ*dnaK*, *dnaK* mutant; *dnaK* comp, *dnaK*-complemented strain; Δ*clpB*, *clpB* mutant; *clpB* comp, *clpB*-complemented strain; Δ*groESL*, *groESL* mutant; *groESL* comp, *groESL*-complemented strain; Δ*flaA*, *flaA* mutant; *flaA* comp, *flaA*-complemented strain; Δ*flaB*, *flaB* mutant; Δ*cheY*, *cheY* mutant; Δ*ruvC*, *ruvC* mutant; *ruvC* comp, *ruvC*-complemented strain; Δ*cmeC*, *cmeC* mutant; *cmeC* comp, *cmeC*-complemented strain; Δ*cmeF*, *cmeF* mutant; *cmeF* comp, *cmeF*-complemented strain; Δ*fur*, *fur* mutant; *fur* comp, *fur*-complemented strain.

^2^Changes of at least fourfold are indicated in bold.

**Supplementary Table 2. Primers used in this study.**

| **Primer** | **Sequence (5’-3’)^1^** | **Purpose** | **Reference** |
| --- | --- | --- | --- |
| **Amplifying antibiotic cassette** | | | |
| Kan-F | GCGATGAAGTGCGTAAG | For amplification of *aphA3* (kanamycin resistance) cassette | (Kim et al., 2023) |
| Kan-R | CGGCTCCGTCGATACTATG |  |  |
| cat-phospho-F | TGCTCGGCGGTGTTCCTTT | For amplification of *cat* (chloramphenicol resistance) cassette | This study |
| cat-phospho-R | GCGCCCTTTAGTTCCTAAGG |  |  |
| **Construction of mutant strains** | | | |
| dnaK-SalI-F | AAAGTCGACGCTATAGAAGCAATGAAGAAAGAG | For amplification of *dnaK* with flanking region | This study |
| dnaK-BamHI-R | AAAGGATCCCCATCTCCCCTACTTGAACTG |  |  |
| dnaK-inv-F | GCGAAGTTTCTCATAAGTTAGCC | For inverse PCR amplification of *dnaK*-cloned pUC19 |  |
| dnaK-inv-R | CGTTCATACACAGCAACACAAGA |  |  |
| clpB-SalI-F | TTTGTCGACGCTTTATCTGCTGGTGTTCCTT | For amplification of *clpB* with flanking region |  |
| clpB-BamHI-R | AAAGGATCCGTATGCGTCAACCTGGAAGCT |  |  |
| clpB-inv-F | CGATATGATACTTGCTGATGAACTT | For inverse PCR amplification of *clpB-*cloned pUC19 |  |
| clpB-inv-R | GAATGTATAGCTAAAGATGCTGCA |  |  |
| groESL-SalI-F | TTTGTCGACGCAATAGGGCTGTAATTATACATTC | For amplification of *groESL* with flanking region |  |
| groESL-BamHI-R | AAAGGATCCGCTAGCAATGAAGAAGTAAATACTA |  |  |
| groESL-inv-F | CGATCCTGTTAAAGTAGAAAGAGTA | For inverse PCR amplification of *groESL*-cloned pUC19 |  |
| groESL-inv-R | CTAGAACACGCTTTCCTAAAGG |  |  |
| cheY-SalI-F | AATGTCGACTGTAGCAAAGAATGAGAAGGTTG | For amplification of *cheY* with flanking region |  |
| cheY-BamHI-R | TATGGATCCCTTTCAATAAGCTTTTTTAACTCAGAA |  |  |
| cheY-inv-F | TAGAACTGTCATCAACAACTAACAA | For inverse PCR amplification of *cheY*-cloned pUC19 |  |
| cheY-inv-R | GAAGTGGAGAAGGTGCAGCT |  |  |
| ruvC-SalI-F | TTTGTCGACGCATGTTGGGTGGATTGTCTG | For amplification of *ruvC* with flanking region |  |
| ruvC-BamHI-R | AAAGGATCCCCCCAAAAAGCAGCAGTTTGATT |  |  |
| ruvC-inv-F | CCACAATTTCTCGAACCTGGATC | For inverse PCR amplification of *ruvC*-cloned pUC19 |  |
| ruvC-inv-R | GGCTTTAACCCATGCAGCAAAT |  |  |
| cmeC-SalI-F | AAAGTCGACTTCAAAACAAAAGCGGAAAAAGCTA | For amplification of *cmeC* with flanking region |  |
| cmeC-BamHI-R | AATGGATCCGGATAGGTTGTGATTTGTTGCG |  |  |
| cmeC-inv-F | ATTTGAGCAAAGTGAAGATACGAG | For inverse PCR amplification of *cmeC*-cloned pUC19 |  |
| cmeC-inv-R | CTGAAATCAAAAGAGTAAAACTTGCT |  |  |
| cmeF-SalI-F | ATTGTCGACATGCAAGATTTAGCGAGAGAAAAAAT | For amplification of *cmeF* with flanking region |  |
| cmeF-BamHI-R | AATGGATCCGTGTTGATTTGATTGATATTTTAGAGG |  |  |
| cmeF-inv-F | CGGTAATAGGTCGGTTTATAGC | For inverse PCR amplification of *cmeF*-cloned pUC19 |  |
| cmeF-inv-R | CTCGTAGTACCTGCACTTTTTAAA |  |  |
| **Confirmation of the mutant strains constructed by natural formation** | | | |
| flaA-con-F | CGATATAGCATTTAACAAGTTCATG | For confirmation of *flaA* gene deletion | This study |
| flaA-con-R | GCAGCTTTTGTAAACTACTGTAG |  |  |
| flaB-con-F | CAAATCCAAGCCTAGTTTAGAACTA | For confirmation of *flaB* gene deletion |  |
| flaB-con-R | CGCTATTTTACCTTTGCTAGACT |  |  |
| **Construction of the complemented strains** | | | |
| dnaK-comp-NotI-F | AAAGCGGCCGCGAGTGGCTCTTATCAAAGATGAAA | For genetic complementation of *dnaK* | This study |
| dnaK-comp-NotI-R | TTTGCGGCCGCGTTTGCTTACACTAAAATGAATTGTCT |  |  |
| clpB-comp-NotI-F | AAAGCGGCCGCGCAATCGTCTAAGTAGAACCATAG | For genetic complementation of *clpB* |  |
| clpB-comp-NotI-R | TTTGCGGCCGCGGAGAAAGTGCTTATTATACCAC |  |  |
| groESL-comp-NotI-F | AAAGCGGCCGCGCACAACAACAAAAGCTACAATG | For genetic complementation of *groESL* |  |
| groESL-comp-NotI-R | TTTGCGGCCGCGGAGGATTTGGTATAGGGCTTT |  |  |
| ruvC-comp-XbaI-F | ATATCTAGAGTTTACAAGCAAGTTCTGTTCCAA | For genetic complementation of *ruvC* |  |
| ruvC-comp-XbaI-R | ATATCTAGATTTTCCAAGTCCTGTAGGTCCA |  |  |
| flaA-comp-XbaI-F | AATTCTAGAAAACTTCATATACAAGATAAAACGCAT | For genetic complementation of *flaA* |  |
| flaA-comp-XbaI-R | TATTCTAGAGATTAAAGCAAAAAGTGTTCCAAGT |  |  |
| cheY-comp-XbaI-F | TTTTCTAGATCAGTTTAGTCGTTTGGTATATTTTTG | For genetic complementation of *cheY* |  |
| cheY-comp-XbaI-R | ATATCTAGACAAGTGCCCATGAAAACTCTTC |  |  |
| cmeC-comp-XbaI-F | TATTCTAGACTTTAGCGATATTCTTTGTGCCTT | For genetic complementation of *cmeC* |  |
| cmeC-comp-XbaI-R | TATTCTAGAGGCTTATGAAATTACAGATGCAGA |  |  |
| cmeF-comp-XbaI-F | ATATCTAGAGCTGAAGTGCAAACCACAAATC | For genetic complementation of *cmeF* |  |
| cmeF-comp-XbaI-R | TAATCTAGAGAAAAAATACAAATCGCCTATGATAAC |  |  |
| fur-comp-XbaI-F | GTGGCCTAGGTTTTTTAGATCG | For genetic complementation of *fur* |  |
| fur-comp-XbaI-R | AAATCTAGAGTCCTGCAACAACAGCATTGA |  |  |
| **qRT-PCR** | | | |
| 16s-RT-F | ATAAGCACCGGCTAACTCCG | For targeting  16S rRNA | (Kim et al., 2023) |
| 16s-RT-R | TTCCATCTGCCTCTCCCTCA |  |  |
| dnaK-RT-F | GGCGAGGTTTTAGTAGGCGA | For targeting  *dnaK* | This study |
| dnaK-RT-R | GCGATTTCTATTGCGCACGC |  |  |
| clpB-RT-F | AGAGCGGGACGAAAAATGGA | For targeting  *clpB* |  |
| clpB-RT-R | ACGCCTGGTTCACCTAAAAGT |  |  |
| groES-RT-F | TCAACCTTTAGGAAAGCGTGT | For targeting  *groES* |  |
| groES-RT-R | TCTGTTCCACCGTATTTAGCA |  |  |
| groEL-RT-F | AACTATGGGGCCAAGAGGAC | For targeting  *groEL* |  |
| groEL-RT-R | TGTTCCATCGCCTGCTTGAT |  |  |

^1^Underlining indicates the enzyme recognition sites.

**References**

Kim, J., Park, M., Ahn, E., Mao, Q., Chen, C., Ryu, S., et al. (2023). Stimulation of surface polysaccharide production under aerobic conditions confers aerotolerance in *Campylobacter jejuni*. *Microbiol. Spectrum* 11(2)**,** e03761-03722. doi: 10.1128/spectrum.03761-22.
